# Supplementary material for: Cold-programmed shape-morphing structures based on grayscale digital light processing 4D printing
Source: Nat Commun. 2023 Sep 8;14:5519. doi: 10.1038/s41467-023-41170-4 (PMC10491591; doi:10.1038/s41467-023-41170-4)
Supplement: Supplementary file 1 — Supplementary Information [file 41467_2023_41170_MOESM1_ESM.pdf]

Cold programmed shape morphing structures based on grayscale digital light processing 4D printing

Liang Yue[1,§], Xiaohao Sun[1,§], Luxia Yu[1], Mingzhe Li[1], S. Macrae Montgomery[1], Yuyang Song[2], Tsuyoshi Nomura[3], Masato Tanaka[2], H. Jerry Qi[1,]*

[1]*The George W. Woodruff School of Mechanical Engineering, Georgia Institute of Technology, Atlanta, GA 30332, USA*

[2] *Toyota Research Institute of North America, Toyota Motor North America, Ann Arbor, Michigan, 48105, USA*

[3]*Toyota Central R&D Laboratories, Inc., Bunkyo-ku, Tokyo 112-0004, Japan*

§These authors contributed equally.

**Corresponding authors, Email: qih@me.gatech.edu*

Supplementary Note 1: Characterizations

The uniaxial tension tests are performed with a universal test machine (Insight 10, MTS Systems Corp., Eden Prairie, MN, USA) with a cross-head speed of 5 mm/min. Dynamic thermomechanical properties are conducted on a DMA machine (Q800, TA Instruments, New Castle, DE, USA) with a temperature ramped at a rate of 10ºC/min. The shape memory behavior of B1 and B2 are conducted by following method. For hot programming, the sample was stretched to 100% with the rate of 10%/min at 100ºC and the temperature was then decreased to 25ºC with the rate of 10ºC/min and held isothermally for 5 mins before remove the load. The recovery was conducted by increase the temperature to 100ºC at 10ºC/min and isothermal for 5 mins. For cold programming, the sample was stretched to 100% with the rate of 10%/min at 25ºC and held for 5 mins before remove the load. The recovery was then conducted by increase the temperature to 100ºC at 10ºC/min. More material properties of the ink and g-DLP printed materials were reported in our previous paper.[1]

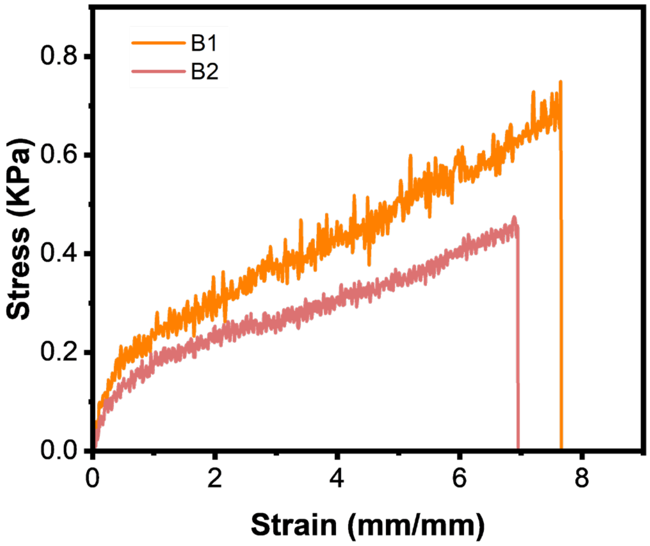

Supplementary Figure 1. Strain-stress curves of B1 and B2 tested at 80 ºC.

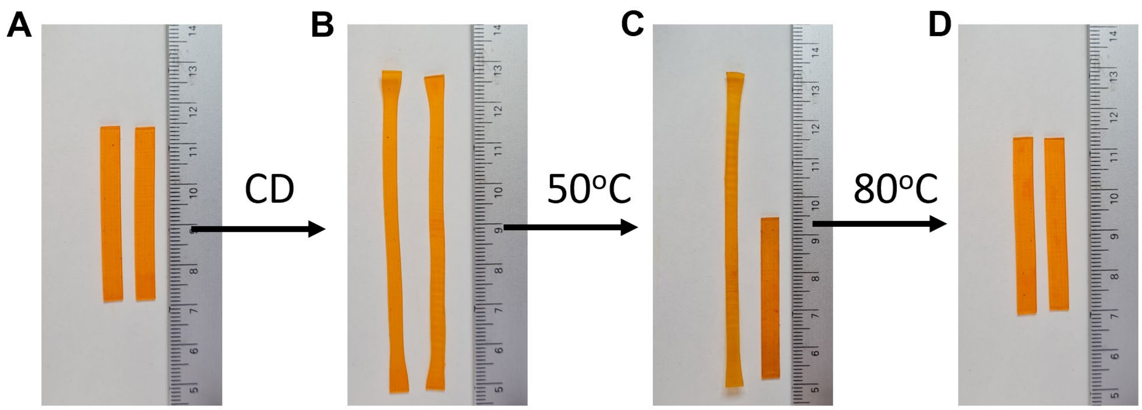

Supplementary Figure 2. (A) B1 and B2 sample as printed; (B) B1 and B2 sample after cold-drawing processing; (C) B1and B2 sample after 50ºC heat treatment, only B2 recovered; (D) after 80ºC heat treatment, B1 recovered. Movie was provided as Supplementary Movie 1.

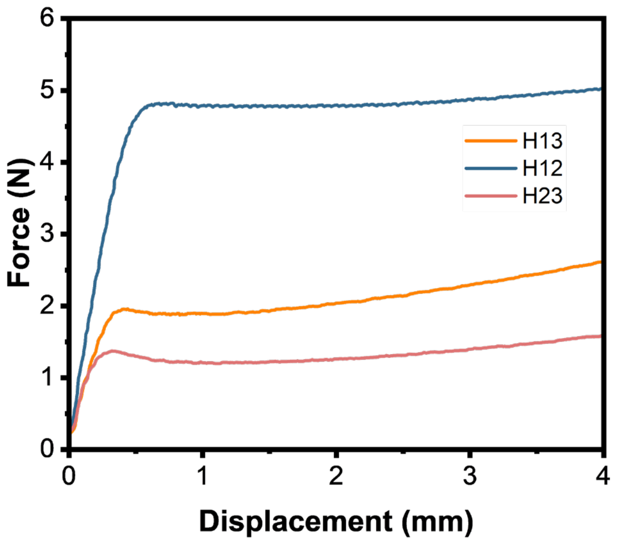

Supplementary Figure 3. Force-displacement curves of the g-DLP printed hinge modules.

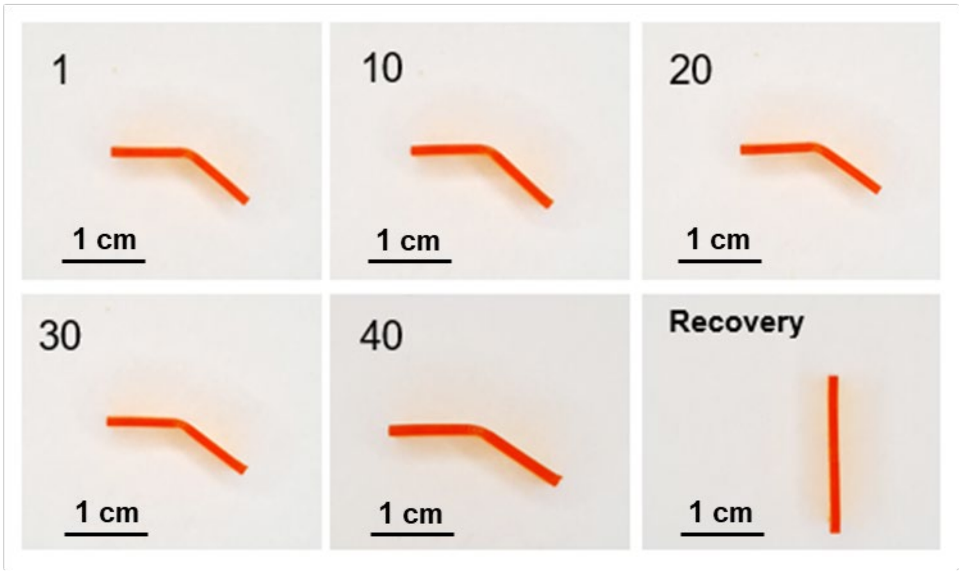

Supplementary Figure 4. H13 hinge cold-draw performance for various cycles and recover to initial state after 40 cycles.

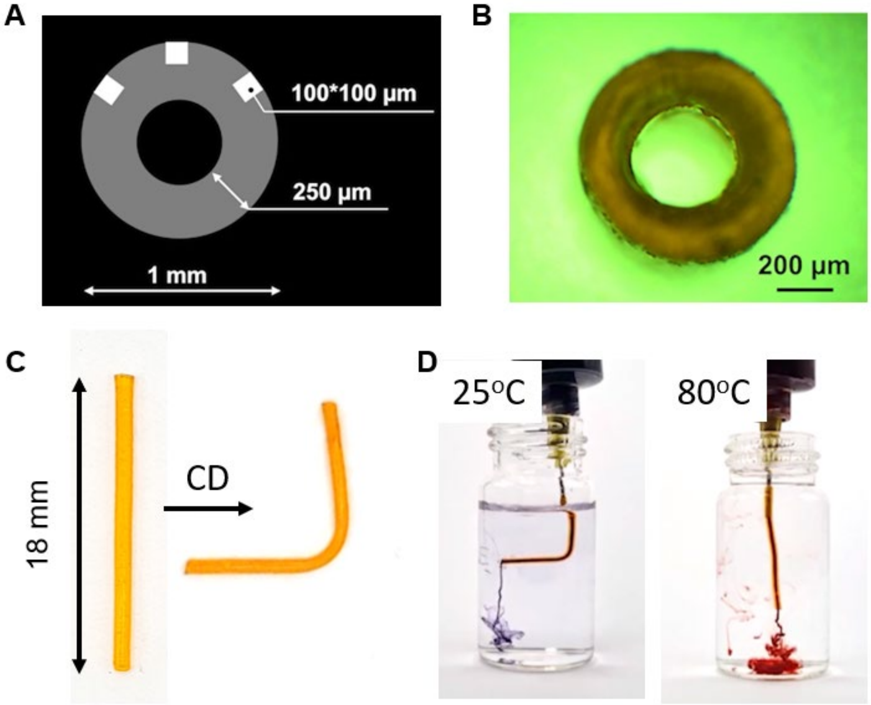

Supplementary Figure 5. (A) Design of hinge structure in a shape morphable pipe; B) the cross section of the printed sample; (C) shape changing of the printed pipe after cold-draw programming; (D) temperature response of the pipe performed in 25 and 80°C. Experimental movie was provided as Supplementary Movie 7.

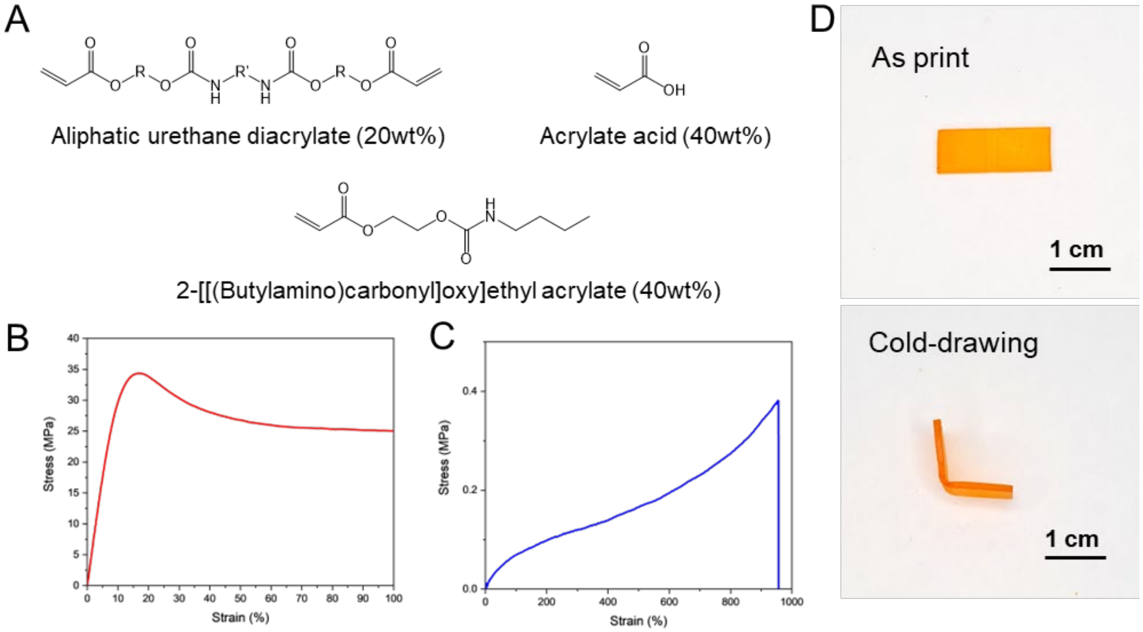

Supplementary Figure 6. (A) Formulation of an alternative g-DLP ink, R and R` represent polyol and diisocyanate segment; strain-stress curves of g-DLP printed with the alternative resin of (B) stiff thermoset state printed with a light intensity of 23.6 mW/cm^2 and (C) rubbery organogel state printed with a light intensity of 3.1 mW/cm^2; (D) cold draw performance of the printed hinge structure.

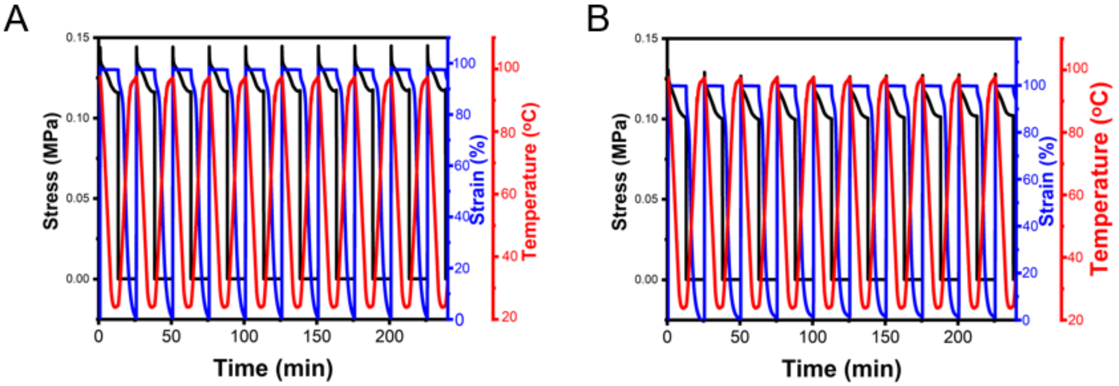

Supplementary Figure 7. Shape memory behaviors of (A) B1 and (B) B2 for ten cycles.

Supplementary Note 2: Finite element modeling

2.1 Constitutive model

The rubbery material B3 is modeled as an incompressible neo-Hookean solid as it is elastic in the studied temperature range (25-100 ºC). The cold-draw and shape memory behaviors of the glassy material (B1 and B2) are captured using an incompressible, stress- and temperature-dependent, multi-branch viscoelastic model, which consists of one equilibrium branch (neo-Hookean model assumed) and multiple nonequilibrium branches (modeled by visco-hyperelastic Maxwell elements) in parallel. In this model, the Cauchy stress **σ** is

$$\boldsymbol{\sigma}(t) = -p\mathbf{I} + \boldsymbol{\sigma}_{ins}^{D}(\mathbf{b}_t) + \operatorname{dev}\left[\mathbf{F}_t\left(\int_0^t \dot{\zeta}\left(\int_s^t \frac{d\xi}{\alpha(\xi)}\right)\mathbf{F}_s^{-1}\boldsymbol{\sigma}_{ins}^{D}(\mathbf{b}_s)\mathbf{F}_s^{-T}\frac{ds}{\alpha(s)}\right)\mathbf{F}_t^T\right] \quad (1)$$

where **F** is the deformation gradient, $\mathbf{b} = \mathbf{F}\mathbf{F}^T$ is the left Cauchy-Green tensor, the subscript "*t*" or "*s*" denotes the deformation from time 0 to *t* or *s*. $\boldsymbol{\sigma}_{ins}^{D}$ is the instantaneous Cauchy stress due to mechanical deformations and follows the neo-Hookean strain-energy potential. $\operatorname{dev}(\bullet) \equiv (\bullet) - (1/3)(\mathbf{I}:(\bullet))\mathbf{I}$ is the deviatoric operator. *p* is an undetermined pressure depending on the boundary conditions. $\zeta(t)$ is the relaxation modulus ratio following the Prony series

$$\zeta(t) = g_{eq} + \sum_{i=1}^{n} g_i \exp\left(-\frac{t}{\tau_i^R}\right), \dot{\zeta}(t) = -\sum_{i=1}^{n} \frac{g_i}{\tau_i^R}\exp\left(-\frac{t}{\tau_i^R}\right)$$
$$\text{with } g_{eq} = \frac{E_{eq}}{E_{ins}}, g_i = \frac{E_i}{E_{ins}}, E_{ins} = E_{eq} + \sum_{i=1}^{n} E_i \quad (2)$$

where E_{eq} is the modulus of the equilibrium branch, E_i the instantaneous modulus of branch *i*, E_{ins} the total instantaneous modulus; they are taken to be small-stress moduli and assumed to be temperature-independent. τ_i^R denotes a reference relaxation time of the branch *i*.

The shift factor, $\alpha(t)$, written as a function of time t in Eq.(1) for brevity, is taken to not only depend on temperature but also stress at time t. Therefore, α can be written as[2]

$$\alpha = \alpha_T \alpha_S \tag{3}$$

where α_T denotes the temperature-dependent shift factor, α_S the stress-dependent shift factor.

The temperature-dependent shift factor α_T is taken to follow the Williams-Landel-Ferry (WLF) approximation for $T>T_{ref}$, i.e.,

$$\lg(\alpha_T) = -\frac{C_1(T-T_{ref})}{C_2+(T-T_{ref})}, \tag{4}$$

and the Arrhenius form for $T<T_{ref}$, i.e.,

$$\ln(\alpha_T) = -\frac{AF_c}{k_b}\left(\frac{1}{T}-\frac{1}{T_{ref}}\right). \tag{5}$$

In Eqs.(4)-(5), C_1, C_2, and AF_c/k_b are material constants, T is the current temperature, T_{ref} represents a structural transition temperature. T and T_{ref} are in Kelvin scale. Note that $\alpha_T = 1$ at $T = T_{ref}$.

The stress-dependent shift factor α_S is taken to follow[2]

$$\ln(\alpha_S) = -\frac{E_S}{k_b T}\frac{M}{s} \tag{6}$$

$$\text{with } M = \sqrt{\frac{1}{2}\mathbf{M}':\mathbf{M}'} \tag{7}$$

where M is the equivalent shear stress, **M** is the Mandel stress, **M**'=dev(**M**), E_S is the activation energy, and s is the athermal shear strength. To capture the softening effects, s evolves through

$$\dot{s} = h_0\left(1-\frac{s}{s_{sat}}\right)\dot{\gamma}^v,\ s = s_0 \text{ when } \gamma^v = 0, \tag{8}$$

where s_0 is the initial value of s, s_{sat} is the saturation value of s, h_0 is a prefactor, γ^v is the

viscous strain, and $\dot{\gamma}^v$ is the viscous flow rate. For a specific branch, $\dot{\gamma}_i^v = M/(\mu_i \tau_i)$, with μ_i denoting the instantaneous shear modulus and $\tau_i = \alpha \tau_i^R$ the relaxation time. Thus, τ_i^R can be interpreted as the relaxation time under small stress and at the reference temperature T_{ref}. Here, to capture essential physics while facilitating the numerical implementation, we use

$$\dot{\gamma}^v = \frac{M}{\mu_{ins} \alpha \tau_0} \tag{9}$$

to define an effective viscous flow rate, instead of that of a specific branch. $\mu_{ins}=E_{ins}/3$ due to the incompressibility. τ_0 is treated as a fitting parameter.

2.2 Numerical implementation

We implement the material model into Abaqus through the built-in viscoelastic model in which the Prony-series and user-defined shift factor can be specified. The Prony-series parameters are specified as those under small stress and at the reference temperature T_{ref} (at which α=1). The shift factor Eq.(3) is implemented through user subroutines USDFLD and UTRS. USDFLD is invoked to access stress values of an integration point and update α_S following Eqs.(6)-(9), which will be passed into UTRS to update α_T and finally α. The updates are in an explicit (or forward Euler) manner.

We also implement the material model into a MATLAB script for the uniaxial tension to assist the parameter identification. We derive the following integration algorithm for the stress updates. The stress at an arbitrary time t, i.e., Eq.(1), can be rewritten as

$$\begin{gathered} \boldsymbol{\sigma}(t) = -p\mathbf{I} + \boldsymbol{\sigma}_{ins}^D(\mathbf{b}_t) - \operatorname{dev}\left[\sum_{i=1}^{n} \frac{g_i}{\tau_i^R} \mathbf{T}_{2i}(t)\right] \\ \text{with } \mathbf{T}_{2i}(t) = \mathbf{F}_t \left(\int_0^t \exp\left(-\int_s^t \frac{d\xi}{\alpha(\xi)\tau_i^R} \right) \mathbf{F}_s^{-1} \boldsymbol{\sigma}_{ins}^D(\mathbf{b}_s) \mathbf{F}_s^{-T} \frac{ds}{\alpha(s)} \right) \mathbf{F}_t^T \end{gathered} \tag{10}$$

Then, the stress at $t+\delta t$ can be updated as

$$\boldsymbol{\sigma}(t+\delta t) = -p\mathbf{I} + \boldsymbol{\sigma}_{ins}^{D}(\mathbf{b}_{t+\delta t}) - \operatorname{dev}\left[\sum_{i=1}^{n} \frac{g_i}{\tau_i^R} \mathbf{T}_{2i}(t+\delta t)\right] \tag{11}$$

where

$$\mathbf{T}_{2i}(t+\delta t) = \exp\left(-\frac{\delta t'}{\tau_i^R}\right)\delta\mathbf{F}\mathbf{T}_{2i}(t)\delta\mathbf{F}^T + \frac{\delta t}{2\alpha(t)}\begin{bmatrix} \exp\left(-\dfrac{\delta t'}{\tau_i^R}\right)\delta\mathbf{F}\boldsymbol{\sigma}_{ins}^{D}(\mathbf{b}_t)\delta\mathbf{F}^T \\ +\dfrac{\alpha(t)}{\alpha(t+\delta t)}\boldsymbol{\sigma}_{ins}^{D}(\mathbf{b}_{t+\delta t}) \end{bmatrix} \tag{12}$$

$$\delta t' = \int_t^{t+\delta t} \frac{dx}{\alpha(x)} \approx \frac{\delta t}{2}\left(\frac{1}{\alpha(t)} + \frac{1}{\alpha(t+\delta t)}\right), \delta\mathbf{F} = \mathbf{F}_{t+\delta t}\mathbf{F}_t^{-1}$$

The trapezoidal integration rule has been used to derive Eq.(12).

2.3 Parameter identification

The SMP parameters including E_{eq}, E_i, τ_i^R, C_1, C_2, and AF_c/k_b are identified using DMA results from multi-temperature and multi-frequency tests.[3] Temperature ramps from 10°C to 130°C in 5°C increment. At each temperature, the material is kept isothermal and stress-free for 5 min and then subjected to a cyclic loading at multiple frequencies (0.1Hz~20Hz). The SMP parameters are identified in two steps. First, the storage modulus E' versus test frequency ω data at different temperatures can be shifted to a master curve at T_{ref}, obtaining discrete data points of $\alpha(T)$. Performing nonlinear numerical fitting to these data points using Eqs.(4)-(5) yields the material constants C_1, C_2, and AF_c/k_b (see **Supplementary Figure 8a** for B1 and **9a** for B2). Second, with the multi-branch model, the storage modulus E' and loss modulus E'' can be written as

$$E' = E_{eq} + \sum_{i=1}^{n} \frac{E_i\left[\omega\tau_i^R\alpha(T)\right]^2}{1+\left[\omega\tau_i^R\alpha(T)\right]^2} \tag{13}$$

$$E'' = \sum_{i=1}^{n} \frac{E_i \omega \tau_i^R \alpha(T)}{1+\left[\omega \tau_i^R \alpha(T)\right]^2} \tag{14}$$

Using Eqs.(13) and (14), performing nonlinear numerical fitting to the shifted E' and E'' versus ω data yields the material constants E_{eq}, E_i, τ_i^R (see **Supplementary Figure 8b** for B1 and **9b** for B2). The obtained parameters are listed in **Supplementary Table 1** and **2**.

The cold-draw parameters s_0, s_{sat}, h_0, τ_0, and E_S/k_b are identified using uniaxial tensile data for different strain rates at room temperature. The parameters are roughly estimated following the procedure of [2] and further optimized by numerical fitting. The obtained parameters lead to simulated stress-strain curves in good agreements with the experiments at multiple strain rates for the two glassy materials (**Supplementary Figure 10**). The obtained parameters are listed in **Supplementary Table 1** and **2**.

All parameters identified above are further used to simulate a complete process of the cold-drawing (i.e., stretching, holding, releasing) and heat-recovering. The simulated stress and strain curves, along with the temperature history, achieve good agreements with the experiments for the two glassy materials (**Supplementary Figure 11**).

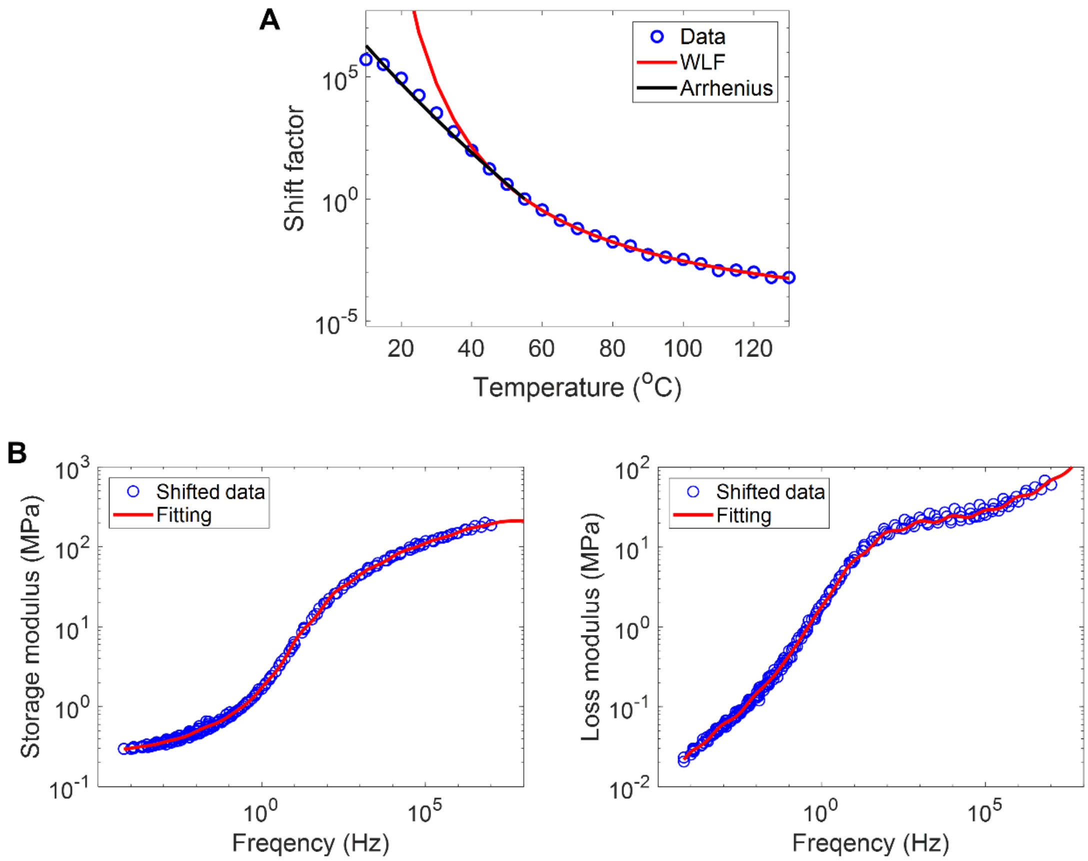

Supplementary Figure 8. SMP parameter identification for the B1 material. Numerical fitting of (A) shift factor versus temperature and (B) storage modulus and loss modulus versus test frequency.

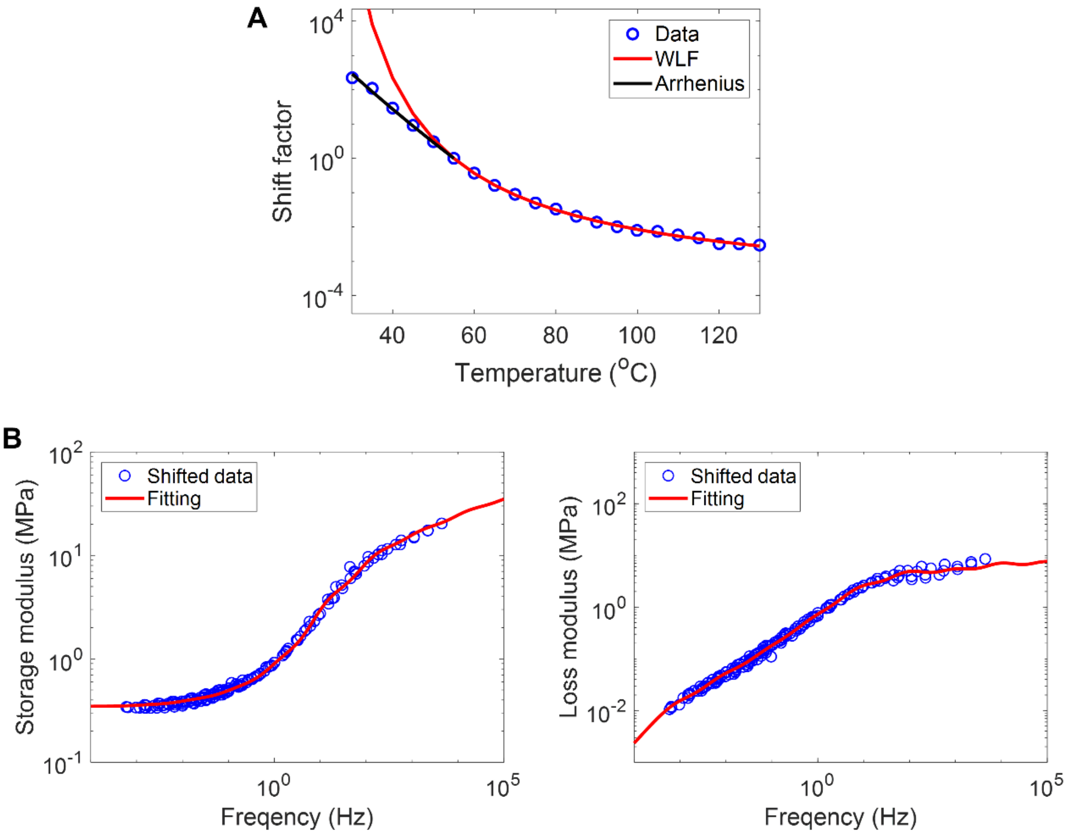

Supplementary Figure 9. SMP parameter identification for the B2 material. Numerical fitting of (A) shift factor versus temperature and (B) storage modulus and loss modulus versus test frequency.

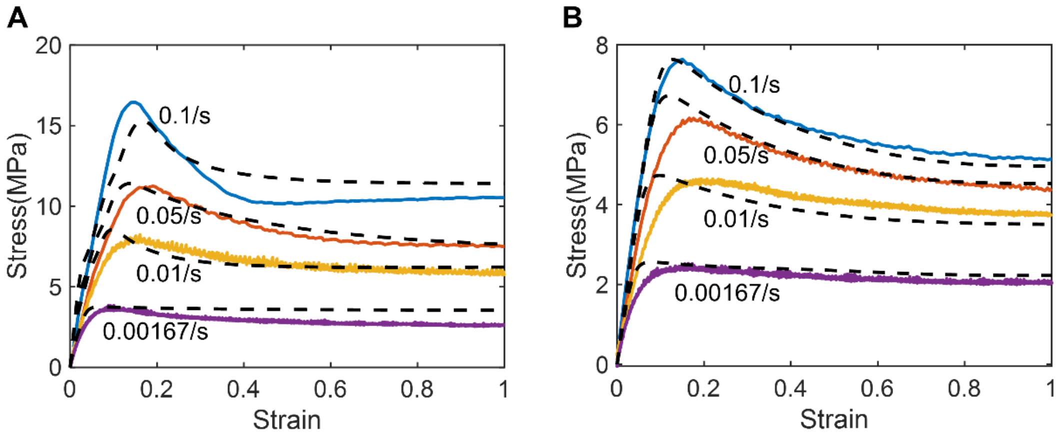

Supplementary Figure 10. Cold-draw parameter identification for the glassy materials. Experimental (solid lines) and FE simulated (dashed lines) uniaxial tensile stress versus strain curves under multiple strain rates for the (A) B1 and (B) B2 materials.

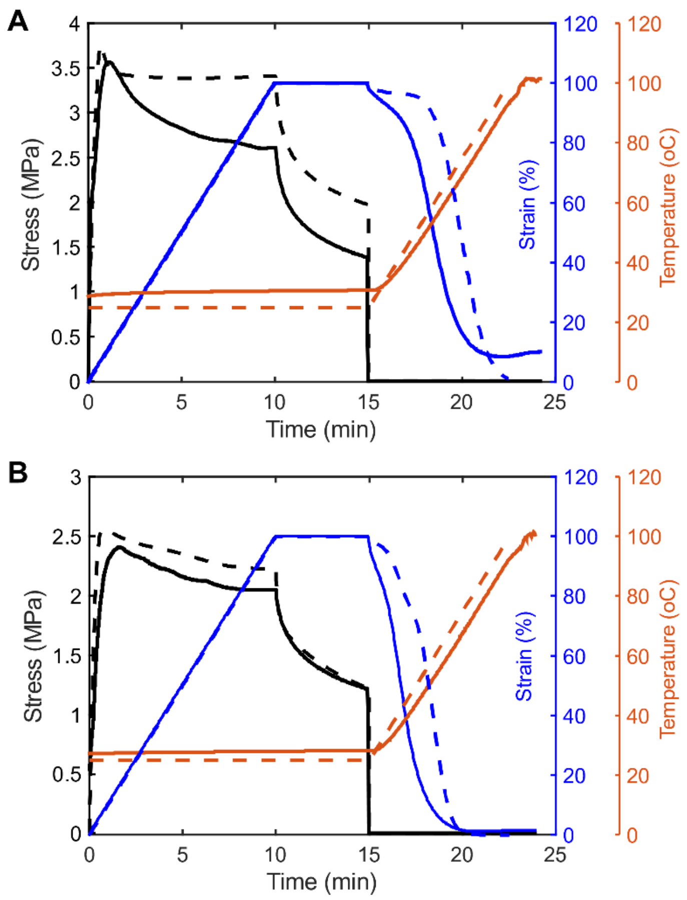

Supplementary Figure 11. Experimental (solid lines) and FE simulated (dashed lines) cold-draw and subsequent heat-recover behaviors for the (A) B1 and (B) B2 materials.

Supplementary Table 1. Material parameters for the B1 material.

Parameters for shift factor		Parameters for branches	
T_{ref} (ºC)	55	E_{eq} (MPa) 0.0704	
C_1	5.62		
C_2 (ºC)	54.77	E_i (MPa)	τ_i^R (s)
AF_c/k_b	-29867	36.170	1.00E-07
E_S/k_b	4400	47.632	1.00E-06
h_0	80.9	34.905	1.00E-05
s_0	16.9	36.126	0.0001
s_{sat}	9.0	25.827	0.001
τ_0	7.2	21.716	0.01
		7.6610	0.1
		0.2406	0.33
		1.2201	1
		0.1498	3.33
		0.3144	10
		0.1895	100
		0.0727	1000
		0.0389	10000
		0.0177	10000000
		0.1951	100000000

Supplementary Table 2. Material parameters for the B2 material.

Parameters for shift factor		Parameters for branches	
T_{ref} (ºC)	55	E_{eq} (MPa) 0.0704	
C_1	3.92	E_i (MPa)	τ_i^R (s)
C_2 (ºC)	55.08		
AF_c/k_b	-22612	10.23584641	1.00E-07
E_S/k_b	2422	10.23151324	1.00E-06
h_0	76.7	10.86287138	1.00E-05
s_0	13.6	10.26097638	0.0001
s_{sat}	6.0	7.204209549	0.001
τ_0	1.57	7.475551187	0.01
		3.478733479	0.1
		0.555568082	1
		0.041392309	3.33
		0.125989885	10
		0.010127193	33.33
		0.049276277	100
		0.015853068	1000
		0.190213505	100000000

Supplementary Note 3: Derivation of hinge folding angle

The folding angle (θ) of the hinge depends on the mechanical properties and geometric designs of two constituent materials (i.e., hinge dimension, fiber position and dimension, etc.). To obtain the folding angle, we first derive the bending curvature using the Euler-Bernoulli beam theory. Consider a composite hinge consisting of glassy fibers and a rubbery matrix (**Supplementary Figure 12**). After stretch and release, let ε_T

denote the temporarily fixed strain of the glassy fiber due to stretch, which is the mismatch strain between two material phases. Then the longitudinal strain (perpendicular to cross-section) for an arbitrary point of position y can be written as

$$\begin{aligned} \varepsilon_f &= -\varepsilon_T + \varepsilon_{NP} - \kappa y' \\ \varepsilon_m &= \varepsilon_{NP} - \kappa y' \end{aligned} \tag{15}$$

where ε_{NP} is the longitudinal strain of a neutral plane (NP), representing the in-plane stretching due to balance of forces; $\kappa y'$ represents the bending strain due to balance of moments, with κ denoting the curvature of NP (positive value means curling up) and $y'=y-y_{NP}$ the relative position to NP (position y_{NP}). It is seen that NP has zero longitudinal strain due to bending. The balance laws for forces and moments are

$$\begin{aligned} &\int_{A_f} E_f \varepsilon_f dA + \int_{A_m} E_m \varepsilon_m dA = 0 \\ &\int_{A_f} E_f \varepsilon_f y' dA + \int_{A_m} E_m \varepsilon_m y' dA = 0 \end{aligned} \tag{16}$$

where E_f and E_m are the Young's modulus of the fiber and matrix, respectively.

Inserting (15) into (16) gives the curvature κ

$$\kappa = \frac{E_m A_m \left(\overline{y}_m - \overline{y}_f\right) \varepsilon_{NP}}{E_f I_{NP-f} + E_m I_{NP-m}}. \tag{17}$$

with relevant variables given by

$$\varepsilon_{NP} = \frac{E_f A_f}{E_f A_f + E_m A_m} \varepsilon_T \tag{18}$$

$$\overline{y}_f = \frac{\int_{A_f} y dA}{A_f}, \overline{y}_m = \frac{\int_{A_m} y dA}{A_m} \tag{19}$$

$$I_{NP-f} = \int_{A_f} \left(y - y_{NP}\right)^2 dA, I_{NP-m} = \int_{A_m} \left(y - y_{NP}\right)^2 dA \tag{20}$$

$$y_{NP} = \frac{E_f A_f \overline{y}_f + E_m A_m \overline{y}_m}{E_f A_f + E_m A_m} \tag{21}$$

$$A_f = w_f t_f, A_m = w_0 t_0 - w_f t_f \tag{22}$$

where ε_{NP} is the axial strain (perpendicular to cross-section) of a neutral plane due to force balance. A_f and A_m are the total area of the fiber and matrix in the cross-section, $\bar{y}_f$ and $\bar{y}_m$ the corresponding area-mean y position, and $I_{NP\text{-}f}$ and $I_{NP\text{-}m}$ the second area moment of the fiber and matrix with respect to the neutral plane, respectively. w_f, w_0, t_f, and t_0 are geometric parameters as shown in **Supplementary Figure 12**. With the help of (18)-(22), κ can be analytically evaluated using (17).

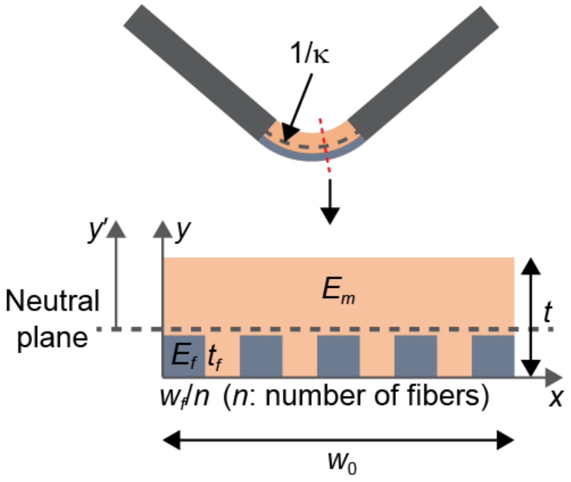

Supplementary Figure 12. Schematic of a bent hinge (top) and a general cross-section design (bottom).

Neglecting the boundary effects between the hinge and panels, the folding angle can be given by

$$\theta = \kappa l_H \left(1 + \varepsilon_{NP}\right) \tag{23}$$

where l_H is the initial length of hinge.

The analytical prediction of folding angle relies on ε_T as an explicit function of applied strain ε_p and visco-plasticity of the material, which is hard to obtain due to the complex constitutive behavior of the glassy material. However, for the glassy material used here, an empirical form is easily obtained by fitting the experimental data, i.e.,

$\varepsilon_T = \varepsilon_P \cdot r_T$, with $r_T = -0.27\varepsilon_P^2 + 0.51\varepsilon_P + 0.71$ being the fixity as shown in Figure 2C of main text. Using this empirical formula and Eqs.(23), the theoretical prediction of the strain-angle relation achieves good agreement with FEA and experiment (**Figure 2C** of main text).

Supplementary References

1. Yue, L., et al., *Single-vat single-cure grayscale digital light processing 3D printing of materials with large property difference and high stretchability.* Nature Communications, 2023. **14**(1): p. 1251.
2. Westbrook, K.K., et al., *A 3D finite deformation constitutive model for amorphous shape memory polymers: A multi-branch modeling approach for nonequilibrium relaxation processes.* Mechanics of Materials, 2011. **43**(12): p. 853-869.
3. Roach, D.J., et al., *4D Printed Multifunctional Composites with Cooling-Rate Mediated Tunable Shape Morphing.* Advanced Functional Materials, 2022. **32**(36): p. 2203236.
