# Supplementary material for: Cold-programmed shape-morphing structures based on grayscale digital light processing 4D printing
Source: Nat Commun. 2023 Sep 8;14:5519. doi: 10.1038/s41467-023-41170-4 (PMC10491591; doi:10.1038/s41467-023-41170-4)
Supplement: Supplementary file 3 — Description of Additional Supplementary Files [file 41467_2023_41170_MOESM3_ESM.pdf]

Description of Additional Supplementary Files

Supplementary Movie 1: Cold-draw programming and recovery

Supplementary Movie 2: Cold-programmed wave structure

Supplementary Movie 3: Cold-programmed helices structure

Supplementary Movie 4: Cold-programmed hand structure

Supplementary Movie 5: Cold-programmed dual-direction panel structure

Supplementary Movie 6: Two-stage cold-programmed shape morphing

Supplementary Movie 7: Cold-programmed straw with micro-hinge

Supplementary Movie 8: g-DLP printing of hinge structure
